# Relationship between Cd and Zn concentration in the kidneys, liver, and muscles of moose (*Alces alces*) from north-eastern Poland

**DOI:** 10.1007/s11356-016-7804-9

**Published:** 2016-10-14

**Authors:** Michał Skibniewski, Ewa M. Skibniewska, Tadeusz Kośla, Katarzyna Olbrych

**Affiliations:** 1 Faculty of Veterinary Medicine, Department of Morphological Sciences, Warsaw University of Life Sciences-SGGW, Nowoursynowska 159 C, 02-776 Warsaw, Poland; 2 Faculty of Animal Sciences, Department of Biology of Animal Environment, Warsaw University of Life Sciences-SGGW, Ciszewskiego 8, 02-786 Warsaw, Poland

**Keywords:** Moose, (*Alces alces*), Wildlife, Cadmium, Zinc, Kidneys, Liver, Muscles

## Abstract

The aim of the study was to evaluate the cadmium and zinc content in the kidneys and liver of moose from north-eastern Poland. Animals were divided with respect to their age. The mean concentration of cadmium in the kidneys of moose studied was 11.31 mg kg^−1^, while in the liver it amounted to 2.68 mg kg^−1^. Age had a significant effect on the content of cadmium in both organs. In the muscles of most animals studied, the cadmium concentrations were below the detection limit. Elevated concentrations were found in three individuals only. Older animals had over six times higher concentrations of cadmium in both kidneys and liver than younger individuals. The cadmium content in kidneys increased with animals’ age while no such relationship was found for zinc. Although older animals had higher mean concentrations of zinc in kidneys, liver, and muscles, the two age groups did not differ significantly. The mean concentration of zinc in the kidneys of moose studied was 38.83 mg kg^−1^, while in the liver it amounted to 29.03 mg kg^−1^. The cadmium concentration in the kidneys was significantly correlated with the cadmium concentration in the liver (*r* = 0.53, *p* ≤ 0.01) and with the zinc concentration in the kidneys (*r* = 0.52, p ≤ 0.01). The data obtained within study correspond with analyses results of the organs of healthy moose in Sweden.

## Introduction

Free-living animals play an important role in environmental assessments of pollution by heavy metals like cadmium, which is a toxic element most widespread in the environment. It occupies the 8th place in the ranking of the 20 most harmful substances (Thirumoorthy et al. 2011). An increase of distribution of this metal due to human activity is now being observed. Because of a high mobility and a tendency for bioaccumulation, the cadmium concentration gradually increases along the food chain. Due to its toxic properties and being widespread in the environment, cadmium focuses the attention of many research teams studying environmental pollution. After ingestion with food by herbivores, the metal is accumulated in the organs such as the kidneys and liver (Danielsson and Frank 2009). Apart from plant tissues, cadmium may be taken up with dust settling on green plant parts (Kośla et al. 2008). Chronic exposure to cadmium results in an irreversible tubular nephropathy, which may develop kidney insufficiency (Eisler 1985; Venäläinen et al. 2005; Thirumoorthy et al. 2011). Zinc is one of the anti-oxidative metals that support the activity of anti-oxidation enzymes (Tubek et al. 2008). The metal is able to reduce the cellular concentration of cadmium and its sequestration by cadmium-induced metallothionein (Kaji et al. 1992; Muller et al. 1994; Antila et al. 1996). Zinc is also a component of many proteins and an activator of more than 300 enzymes including lactate dehydrogenase, alkaline phosphatase, and carbonate anhydrase. The element takes part in the metabolism of proteins and carbohydrates, in wound healing, in reproduction, in respiration, in visual perception, in proper functioning of the kidneys, and in taste perception (Prasad 2002; Skibniewska et al. 2013). It is also a component of nuclear receptors of steroid and thyroid hormones (Drake and Sky-Peck 1989). An extremely important role of zinc is its contribution to immunological response and its deficit manifest of themselves in general impairment of immunity (Rink and Hasse 2007). Therefore, the competitive activity of cadmium to zinc plays a key role in the origin of many metabolic disorders (Skibniewska et al. 2012).

The assessment of environmental pollution by cadmium was based on analyses of its content in the organs of representatives of the deer family. The moose (*Alces alces,* L. 1758) is a representative of the largest wild ruminants of the northern hemisphere. Being a free-living animal, moose is a good biomarker of environmental pollution by cadmium and its organs are often used in ecotoxicological studies (Custer et al. 2004; Arnold et al. 2006; Danielsson and Frank 2009). No publications exist in the available literature on cadmium concentrations in the organs of moose from mid-eastern Europe. In Poland, the species is protected and a moose-shooting moratorium has been in effect since 2001. For this reason, obtaining appropriate material for studies is extremely difficult.

Recently, the moose population in Poland has increased from 2.1 thousands in 2000 to 15.6 thousands in 2014. In 2010, the number of animals exceeded 8000 (Budny et al. 2010; Central Statistical Office 2015). As a result, the possibility of withdrawing the moose-shooting moratorium for the further shooting seasons is under consideration. Although in the last decades an increase of moose population in Poland can be observed, there is lack of comprehensive information about its general health status, physiology, and trace element concentrations, including essential as well as toxic elements in their tissues. Therefore, the aim of this study is to evaluate the cadmium and zinc concentrations in the parenchymatous organs of moose with regard to the age of the animals studied and to assess the bioavailability of both metals in their relatively unpolluted habitat, which is commonly described as the green lungs of Poland.

## Material and methods

**Fig. 1:**
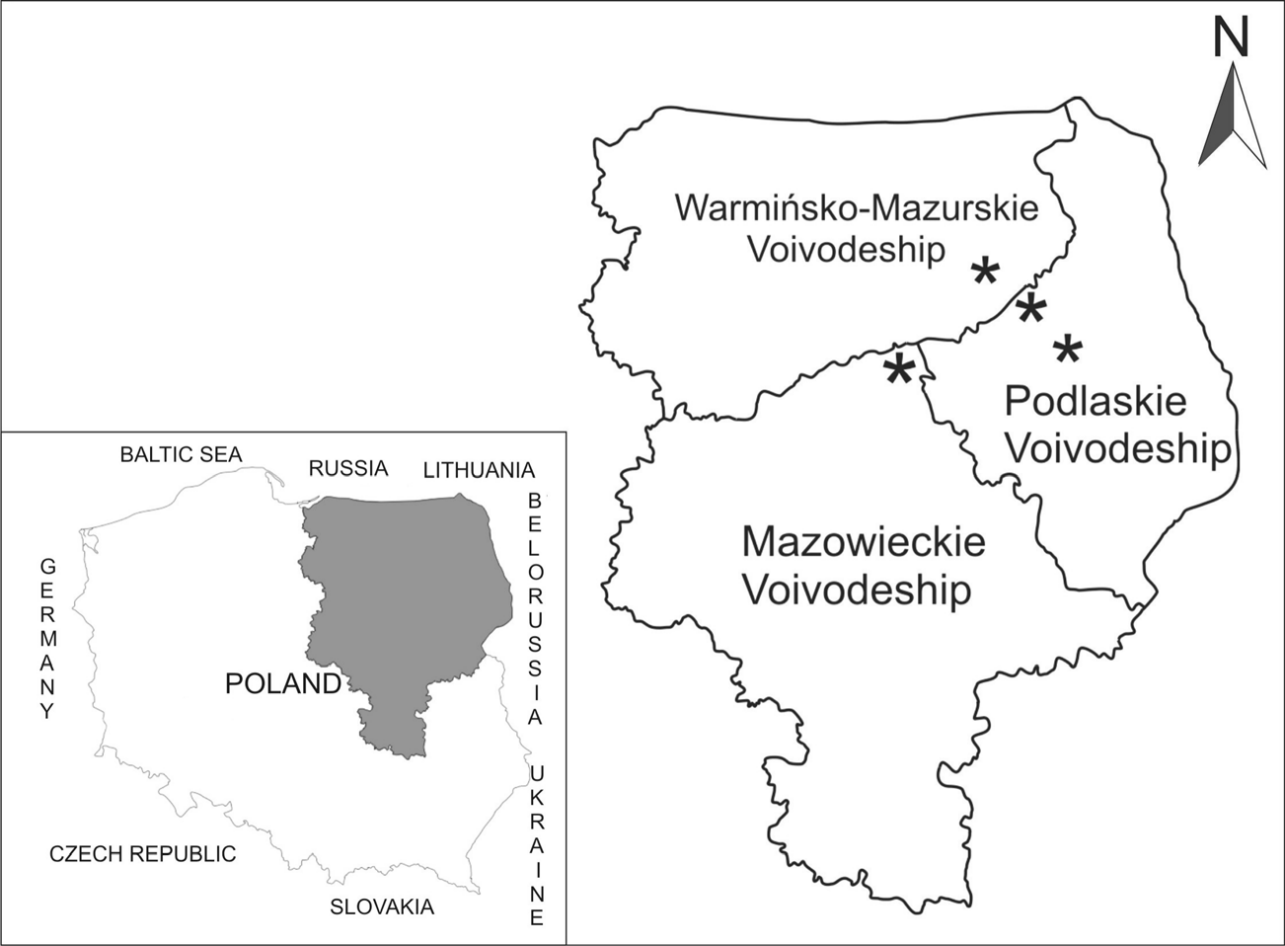
Map of Poland showing areas where samples were collected

The study material consisted of samples of the liver, kidneys, and skeletal muscles of moose from north-eastern Poland. The Minister of Environmental Protection issued a permit for shooting 90 individuals in October and November 2010 for scientific purposes, following an application from the Biology Institute in Białystok (decision number DL.gł-6713-5/45392/10/PJ). After protests by environmental organizations, the permit was withdrawn and finally, material for elemental analysis was obtained from 35 individuals. Selection shooting was performed in places shown in Fig. 1. Material for the study was collected from animals hunted in a few sites located in three provinces of north-eastern Poland. The study sampling areas are represented by regions bordering Russia, Lithuania, and Belorussia. North-eastern Poland is a region of low industrialization with many protected areas, including forests, peatlands, and river valleys, which are typical refuges of moose. More than 70 % of Polish moose live in this part of Poland.

The mean age of animals was 7 years. Body mass varied from 68 to 235 kg with a mean of 171 kg. Animals were divided with respect to their age. The first group comprised animals less than 2 years old, while the second group was comprised of fully mature animals older than 3 years. Kidney samples were prepared in such a way, that they contained the cortex of this organ only. Liver samples had a form of triangles collected from the verge of the right lobe while muscle samples consisted of the sections of *musculus masseter.* The study material comprised sections of both superficial and deep portions of the muscle mentioned above.

After collection, the samples were placed in tight polyethylene bags and frozen at −20 °C. Just before chemical analyses, the samples were homogenized and placed in Teflon vessels. Mineralization was performed in a microwave Milestone system. Cadmium and zinc concentrations were determined with the inductively coupled plasma mass spectrometry (ICP-MS, ELAN DRC II, Perkin Elmer, USA). The accuracy of the method was verified by using certified reference material CRM—BCR 185R (Community Bureau of Reference, BCR in Brussels, Belgium). The percentages of recovery were 118 and 102 for Zn and Cd, respectively. Discrepancies between the certified values and concentrations quantified were below 10 %. All analyses were performed in triplicate. Obtained results are presented as means expressed in milligram per kilogram fresh mass of studied organs. Statistical analysis was performed with Statistica 12.0 software (StatSoft Inc.). Before analyses, the data were tested for normality with Shapiro-Wilk *W* test. Concentrations of both metals were not normally distributed. Therefore, the non-parametric Mann-Whitney *U* test was used to check the significance of differences between groups. Relationships between the concentrations of cadmium and zinc were calculated by using Spearman’s correlation coefficients at *p* ≤ 0.05 and *p* ≤ 0.01.

## Results

Mean concentrations of cadmium and zinc in the kidneys, liver, and muscles of animals are presented in Table 1. Mean concentrations of cadmium in moose kidneys was 11.31 mg kg^−1^, and age had a significant effect on the content of cadmium. Concentrations of this metal were over six times higher in the kidneys of older than in younger individuals. Similarly, older animals had more than six times higher concentrations of cadmium in the liver than the younger ones. In most muscle samples, cadmium concentrations were below the detection limit. In three cases, elevated concentrations were found, namely 1.25, 0.36, and 0.15 mg kg^−1^ wet mass. All animals with high cadmium levels were represented by the group of fully mature individuals.

**Table 1:** Cadmium and zinc concentrations in kidneys, liver and skeletal muscles of the moose

Material	Parameter	Group 1 animals <2 years old *n* = 8		Group 2 animals >3 years old *n* = 27		All animals *n* = 35		M-W *U* test group1 vs. group 2	
		Cd	Zn	Cd	Zn	Cd	Zn	Zn	Cd
Kidney	Median	3.01	30.00	10.94	34.00	7.58	33.00	ns	*p* ≤ 0.01
	AM	2.34	43.00	14.57	37.32	11.31	38.83		
	SD	1.85	34.58	11.83	9.06	11.51	18.83		
	Range	0.10–4.72	22.00–125.00	3.92–56.24	24.00–57.00	0.10–56.24	22.00–125.00		
Liver	Median	0.33	24.50	1.57	27.50	1.09	27.00	ns	*p* ≤ 0.01
	AM	0.52	25.38	3.34	30.15	2.68	29.03		
	SD	0.59	4.53	3.65	8.58	5.87	8.02		
	Range	0.03–1.96	20.00–32.00	0.20–26.90	17.00–49.00	0.03–26.90	17.00–49.00		
Muscles	Median	nd	45.00	nd	45.50	nd	45.00	ns	nd
	AM	nd	43.57	nd	44.61	nd	44.40		
	SD	nd	10.06	nd	14.74	nd	13.80		
	Range	nd	25.00–57.00	nd	16.00–97.00	nd	16.00–97.00		

The values are expressed in mg∙kg^−1^ wet mass. Differences are highly significant at *p* ≤ 0.01

*SD* standard deviation, *AM* arithmetic mean, *ns* non-significant difference, *nd* not detected

No such relationship was found for zinc. The two age groups did not differ significantly.

Data for all animals showed that cadmium concentration in the kidneys was significantly correlated with cadmium concentration in the liver (*r* = 0.53, *p* ≤ 0.01) and with zinc concentration in the kidneys (*r* = 0.52, p ≤ 0.01). Concentrations of cadmium and zinc in the liver were also significantly correlated (*r* = 0.57, *p* ≤ 0.01). Data on the relationships between the concentrations of metals in particular organs are presented in Table 2.

**Table 2:** Correlation coefficients between zinc and cadmium in the organs examined

	Zn kidney	Zn muscle	Cd liver	Cd kidney	Cd muscle
Zn liver	0.43*	−0.41*	0.57**	0.32	−0.10
Zn kidney	–	−0.25	0.32	0.52**	0.26
Zn muscle	–	–	−0.26	0.01	−0.20
Cd liver	–	–	–	0.53**	−0.33
Cd kidney	–	–	–	–	−0.04

**Correlation coefficient significant at *p* ≤ 0.01

*Correlation coefficient significant at *p* ≤ 0.05

## Discussion

### Cadmium

There is no data in the scientific literature on cadmium concentrations in the organism of moose from Poland. This is mainly a result of the special form of protection, established due to the fact that in the last century moose populations in Poland have twice been on the border of extinction. The first dramatic decrease of the animals’ number was in the 40’s. The total number of moose was just a few individuals, all of which inhabited the Biebrza river valley (Steinbach 2009). The second depression of moose populations took place in the 80s and 90s due to excessive hunting. Since the establishment of the moose-shooting moratorium in 2001, its population in Poland has constantly grown. However, in the last few years, growth rate decreases have occurred due to the high density of animals. In certain areas of north-eastern Poland, the number of animals exceeds 15 individuals per 1000 ha, while the optimal population density is 7–8 animals per 1000 ha. Therefore, the possibility of withdrawing the moose-shooting moratorium is under consideration (Ratkiewicz 2011). The animals live not only in forested areas of low-human population density but also in forests surrounding large cities, including the capital of Poland. Moose are an important element in environmental assessments of the risk of pollution in their habitats. Among large free-living herbivores, moose seems to be the most suitable species for monitoring the cadmium levels due to its behavior. The animal lives in a relatively settled way of life, and usually, its migrations do not exceed the distance between 50 and 80 km (Frøslie et al. 1984; Frank et al. 2004).

Cadmium is an extremely mobile element that accumulates in an organism. After ingestion, it is rapidly transferred from blood to target organs, mainly to the liver and kidneys, where it is bound with metallothioneins (Satarug et al. 2003; Bilandžić et al. 2009). Studies of cadmium content in domesticated, wild, and laboratory animals have been carried out for many years, but the proper interpretation of obtained results is difficult (Anke et al. 1989a, b; Kośla et al. 2004, 2008). Of particular importance is the process of sampling and preparation for laboratory determinations. In the analyses of cadmium concentrations in the kidneys, one should pay attention as to whether the sample is taken from the cortex or from the renal medulla. Cadmium is known to accumulate mainly in the cortex; hence, presented data reflect the upper levels of cadmium in the kidneys and should not be directly compared with the metal content in the whole organ (Arnold et al. 2006).

There are few papers devoted to the elemental analysis of tissues of European moose. Most studies were performed in Scandinavian countries where populations of these animals are numerous. In the mid-1980s, a disease of unknown etiology was observed in moose. Numerous changes resembling molybdenosis and copper deficit in cattle and sheep were observed post-mortem in sick animals (Frank 2004). Therefore, studies on the content of essential metals and toxic elements were undertaken in the organs of moose. The material collected in Sweden from healthy animals and from those that showed disease symptoms was analyzed. In healthy moose, the median cadmium concentration in the kidneys was 7.3 mg kg^−1^ fresh mass and in the liver was 1.0 mg kg^−1^ fresh mass. In sick animals, the respective values were 10.95 and 1.86 mg∙kg^−1^ (Frank et al. 2000). Although median values of cadmium concentrations from our study (7.58 mg kg^−1^ in the kidneys and 1.10 mg kg^−1^ in liver) were similar to those obtained from analyses of material collected from healthy individuals in Sweden, results of the previous study suggest that animals from north-eastern Poland may also be threatened by primary copper deficiency (Skibniewski et al. 2016).

In contrast, our results (Table 1) were definitely higher than data presented by Venäläinen et al. (2005) who analyzed samples obtained from animals hunted in four regions of Finland. The highest mean value noted in the kidneys was 6.18 mg kg^−1^, while the lowest was 4.95 mg kg^−1^ wet mass for central and southern Finland, respectively. In the liver samples, the highest mean cadmium concentration was 1.28 and the lowest 0.71 mg kg^−1^ wet mass.

Studies concerning cadmium accumulation in the tissues of moose were also performed in the USA and Canada. The mean concentration of cadmium in the kidneys of animals from north-eastern Poland was higher than in moose from three Alaskan regions, namely from Palmer, Kenai, and Yakutat. In the latter, the concentrations of cadmium in the cortex of moose kidneys were 4.06, 6.57, and 1.68 mg kg^−1^ wet mass, respectively. Only in individuals from Galena the cadmium content was twice that in our study (Arnold et al. 2006). A similar relationship was found for liver samples. Results obtained in our study were higher than those from the three mentioned Alaskan regions and lower than in individuals from Galena. Other authors reported much higher concentrations of cadmium in the kidneys of moose from Alaska. O’Hara et al. (2001) found 21.6 ± 20.8 mg kg^−1^ wet mass of Cd in individuals from the catchment basin of the Colville River and 73.1 mg kg^−1^ in moose from other regions of Alaska. However, one should have in mind that these data pertain to the cadmium content in both cortex and renal medulla and, therefore, cannot be directly compared with our results. The kidneys of moose from Yukon Territory contained on average 28.11 mg kg^−1^ wet mass while the livers on 4.94 mg kg^−1^ (Gamberg et al. 2005). Studies were also carried in moose from other regions of North America. The median content of cadmium in the kidneys and liver of individuals from Nova Scotia was 26 and 7.6 mg kg^−1^ wet mass, respectively (Frank et al. 2004). In our studies, the median concentration of cadmium was 7.58 mg kg^−1^ in the kidneys and 1.10 mg kg^−1^ in the liver. Cadmium concentrations in the kidneys of animals from north-eastern Poland were much lower than in individuals from Nova Scotia. The kidneys of moose from Newfoundland contained on average 5.52 mg kg^−1^ and the livers on 1.04 mg kg^−1^ wet mass (Brazil and Ferguson 1989). Concentrations of cadmium in the kidneys and liver of Polish moose were nearly two times higher. Custer et al. (2004) found that the concentration of cadmium in the liver of animals found dead in north-western Minnesota were 0.7 mg kg^−1^ wet mass in moose from agricultural land and prairie and 0.4 mg kg^−1^ wet mass (both values recalculated from dry mass) in animals living on peatlands and in forests. According to data published by Puls (1994), the cadmium concentrations in the parenchymatous organs of moose varies greatly from 0.2 to 9.0 mg kg^−1^ wet mass in the liver and from 0.2 to 100 mg kg^−1^ wet mass in the kidneys.

An interesting problem is the relationship between the cadmium content in the kidneys and in the liver. The kidneys should be preferred as an indicator organ for cadmium, but in some circumstances only its content in the liver could be estimated. Danielsson and Frank (2009) determined this relationship based on samples from 3763 animals. They found that the cadmium concentrations in the kidneys increase with age faster than in the liver. Therefore, a direct comparison of both organs is difficult and the transfer of results from one to the other does not give reliable effects. We found that older individuals had higher concentrations of cadmium in both kidneys and liver. The difference between age groups was highly significant for the kidneys (*p* ≤ 0.01) and significant for the liver (*p* ≤ 0.05). Based on data obtained by other researchers, it was generally assumed that cadmium concentrations in the kidneys are positively correlated with the age of an individual. This relationship is not so strong for the liver. Similar results were obtained by Medvedev (1999) and Arnold et al. (2006) who used non-parametric data processing.

In case of the skeletal muscles, cadmium levels in most animals studied were below quantification limits. There were only three individuals with elevated concentrations of this toxic element. If the moose-shooting moratorium is withdrawn in the next hunting seasons, their meat probably will be directed to consumption. According to EC (2014) regulations, the maximum tolerable level of cadmium in muscle meats of farm animals, excluding horse, is 0.050 mg kg^−1^ wet mass. This value is definitely higher for horsemeat in which the highest acceptable cadmium level is 0.2 mg kg^−1^ wet mass. Taking into account the above mentioned regulations, the observed concentrations of cadmium in the muscles of young moose do not pose a threat to consumers’ health. However, incidentally noted high cadmium levels in the muscles of older individuals require a careful approach to their suitability as a source of food for humans. This phenomenon may be due to the kind of the muscle analyzed. In animals with the highest cadmium level in the muscle sample (1.24 mg kg^−1^), high concentrations in the liver and kidneys were not observed. Masseter muscle belongs to masticatory musculature with high activity, especially in ruminants. This muscular unit neighbors with the oral cavity and lymphatic structures of the head such as mandibular, retropharyngeal, and parotid lymph nodes. It is therefore possible that the high cadmium concentration in the muscle results from the direct passage via mucous membrane of the oral cavity and lymphatic vessels without influence of the portal system.

In the case of offal for species representing farm animals, the maximal level of cadmium should not exceed 0.5 and 1.0 mg kg^−1^ fresh mass for the liver and kidneys, respectively (EC 2014). The values noted in moose representing both age groups significantly exceed recommendations. Therefore, their edible internal organs should be treated as a waste tissue.

It remains unclear how cadmium ends up to the food chain in relatively unpolluted regions of Poland. One of the reasons may be the low pH of the soils in this area, which does not exceed a value of 5.66. The average cadmium concentration in arable soils of Poland, estimated on the base of representative studies, is 0.21 mg kg^−1^ (Terelak et al. 1997). In the year 2010, median cadmium concentration in Polish soils was 0.17 mg kg^−1^, which was a value slightly lower than in the previous years (Siebielec et al. 2012). Its availability for plants increases with soil acidity. Therefore, it is possible for soils even in relatively unpolluted regions to accumulate cadmium in a soluble form, making it biologically available for plants consumed by ruminants.

### Zinc

Unlike cadmium, zinc concentrations do not change markedly with age of individuals. We did not find significant differences between the studied age groups. In moose from Sweden, the content of zinc in the liver of healthy animals was 54.8 mg kg^−1^ wet mass while in those that showed symptoms of the so-called mysterious moose disease, it was 66.8 mg kg^−1^. In the kidneys of healthy animals, the zinc concentration was 40.6 mg kg^−1^ on average and in sick individuals it was 48.5 mg kg^−1^ (Frank et al. 2000). These values are higher than those obtained in our study. In the liver of moose from Sweden, zinc content was almost two times higher than in individuals from Poland. Mean zinc content in the kidneys of healthy animals was similar. In 422 animals from Norway, the mean zinc content in the liver was 31.4 mg kg^−1^ wet mass (recalculated from dry mass), which was also similar to values recorded in our study (Vikøren et al. 2011). Venäläinen et al. (2005), who analyzed zinc concentrations in moose from different regions of Finland, noted rather stable zinc levels over a long period of time. The highest mean zinc concentration in the liver (32.1 mg kg^−1^ wet mass) was noted in animals from south-eastern Finland, while the lowest (23.7 mg kg^−1^) in animals inhabiting south-western Finland. In the kidneys of Finnish moose, the values observed were almost equal. The highest mean zinc concentration (32.4 mg kg^−1^) was recorded in central regions of country and the lowest (30.0 mg kg^−1^) in the southern part. Material collected from Polish moose contained on average 29.03 mg Zn kg^−1^ in the liver and 38.8 mg Zn kg^−1^ in the kidneys, which was similar to values recorded in Finland.

Comparing the mean zinc concentration in the muscles of animals from Poland to Finnish moose, it can be stated that the results of our study are definitely lower. The lowest mean level reported by Venäläinen et al. (2005) was 56.9 mg kg^−1^ wet mass, whereas the mean concentration of zinc in Polish moose was 44.4 mg kg^−1^. The results obtained in our study varied markedly from 16 to 97 mg kg^−1^ wet mass.

In animals from North America, Frank et al. (2004) observed that median concentrations of zinc in the kidneys and liver of moose from Nova Scotia were 50 and 46 mg kg^−1^, respectively. Custer et al. (2004) reported that the mean concentration of zinc in the kidneys of dead moose from north-eastern Minnesota was 167 mg kg^−1^ dry mass in individuals from agricultural lands and prairie and 219 mg kg^−1^dry mass in animals living on peatlands and in forests (33.4 and 43.8 mg kg^−1^ wet mass, respectively). The mean concentration of zinc in the kidneys of moose from Yukon was 29.24 mg kg^−1^and in the liver was 34.87 mg kg^−1^wet mass (Gamberg et al. 2005). Reported values were similar to those obtained from analyses of material collected from Polish moose.

## Conclusions

Data obtained in our study correspond with results of analyses of the organs of healthy moose in Sweden but were definitely higher than those in animals from North America. It was found that the cadmium content in the kidneys and the liver increased with animals’ age. Although in the majority of the muscle samples cadmium levels were below quantification limits, we found three individuals with elevated concentrations of this toxic element. The results of this study has led us to conclude that even in relatively unpolluted areas of Poland cadmium can enter the food chain and accumulate in the internal organs and muscles of free-living ruminants. The zinc content in the kidneys, liver, and muscles of moose was similar to that noted in this species in other regions of the world.

Despite numerous doubts concerning the moose-shooting moratorium in Poland, there is a lack of comprehensive information about its mineral as well as toxicological profile. We are of the opinion that our research, together with the results obtained by other research teams in area of biology, reproduction and genetics of the moose species, may be a useful aid for decision makers as to whether the moratorium should be withdrawn or not.
